# (Tris{2-[2-(2,3,5,6-tetra­fluoro-4-iodo­phen­oxy)eth­oxy]eth­yl}amine)­potassium iodide

**DOI:** 10.1107/S1600536813010532

**Published:** 2013-04-24

**Authors:** Gabriella Cavallo, Hannes Neukirch, Pierangelo Metrangolo, Tullio Pilati, Giuseppe Resnati, Giancarlo Terraneo

**Affiliations:** a NFMLab, Department of Chemistry, Materials and Chemical Engineering, "G. Natta", Politecnico di Milano, Via Mancinelli, 7, I-20131 Milano, Italy

## Abstract

The title adduct, [K(C_30_H_24_F_12_I_3_NO_6_)]I, gives an extended tape of cations linked through I⋯I^−^ halogen bonds (XBs), two of them being quite short and one quite long. In the structure, the cation is hosted in a cavity formed by the arms of the podand which presents a closed conformation wherein two tetra­fluoro­iodo­benzene rings are near parallel [dihedral angle = 15.8 (4)°; centroid–centroid distance = 3.908 (5) Å] and the third ring is closer to orthogonal [dihedral angles = 66.28 (14) and 75.20 (19)°] to the other two rings. The coordination sphere of the K^+^ cation is composed of the six O atoms, the N atom and an F atom in the *ortho* position of one of the rings.

## Related literature

For the synthesis of tris­{2-[2-(2,3,5,6-tetra­fluoro-4-iodo­phen­oxy)eth­oxy]eth­yl}amine and its NaI adduct, see: Mele *et al.* (2005 ▶). For its HI salt, see: Abate *et al.* (2009 ▶).
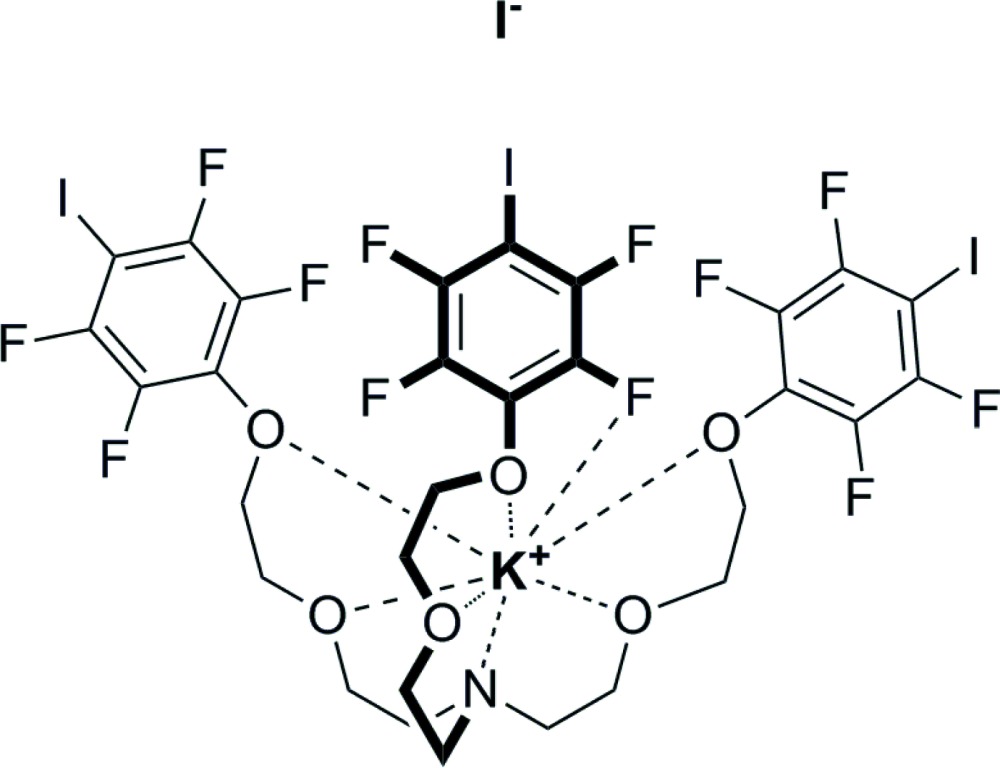


## Experimental

### 

#### Crystal data

[K(C_30_H_24_F_12_I_3_NO_6_)]I
*M* *_r_* = 1269.20
Monoclinic, 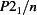

*a* = 13.037 (2) Å
*b* = 23.355 (3) Å
*c* = 13.787 (2) Å
β = 104.83 (3)°
*V* = 4058.0 (11) Å^3^
*Z* = 4
Mo *K*α radiation
μ = 3.27 mm^−1^
*T* = 295 K
0.34 × 0.22 × 0.10 mm

#### Data collection

Bruker SMART APEX diffractometer
Absorption correction: multi-scan (*SADABS*; Bruker, 1998 ▶) *T*_min_ = 0.759, *T*_max_ = 1.000
24956 measured reflections
7254 independent reflections
5627 reflections with *I* > 2σ(*I*)
*R*_int_ = 0.024

#### Refinement

*R*[*F* ^2^ > 2σ(*F* ^2^)] = 0.041
*wR*(*F* ^2^) = 0.119
*S* = 1.06
7254 reflections
487 parameters
H-atom parameters constrained
Δρ_max_ = 2.37 e Å^−3^
Δρ_min_ = −1.25 e Å^−3^

### 

Data collection: *APEX2* (Bruker, 1998 ▶); cell refinement: *SAINT* (Bruker, 1998 ▶); data reduction: *SAINT*; program(s) used to solve structure: *SIR2002* (Burla *et al.*, 2003 ▶); program(s) used to refine structure: *SHELXL2012* (Sheldrick, 2012 ▶); molecular graphics: *ORTEP-3* (Farrugia, 2012 ▶) and *Mercury* (Macrae *et al.*, 2006 ▶); software used to prepare material for publication: *SHELXL2012*.

## Supplementary Material

Crystal structure: contains datablock(s) global, I. DOI: 10.1107/S1600536813010532/pk2473sup1.cif

Structure factors: contains datablock(s) I. DOI: 10.1107/S1600536813010532/pk2473Isup2.hkl

Additional supplementary materials:  crystallographic information; 3D view; checkCIF report

## Figures and Tables

**Table 1:** I⋯I^−^ XBs and short F⋯F contacts (Å and °)

C—*X*⋯*Y*	*X*⋯*Y*	C—*X*⋯*Y*
C8—I1⋯I4	3.4106 (6)	179.30 (13)
C8—I2⋯I4^i^	3.4157 (6)	168.45 (13)
C8—I3⋯I4^ii^	3.9437 (7)	179.21 (18)

Symmetry codes: (i) −*x*, −*y*, −*z*; (ii) −*x*, −*y*, −*z* + 1.Note: for the sake of comparison, a Cambridge Structural Database (Allen, 2002 ▶) search for C—I⋯I^−^ contacts gives 109 hits with a mean I⋯I^−^ distance of 3.53 (3) Å, the lower and higher quartiles being 3.43 and 3.63 Å, respectively.

**Table 2:** Short contacts in receptor–metal cation system for KI and NaI adducts

	K^+^	Na^+^
N1	2.903 (4)	2.515 (15)
O1	2.744 (3)	2.388 (12)
O2	2.849 (3)	2.716 (12)
O3	2.707 (4)	2.463 (13)
O4	2.809 (4)	2.424 (12)
O5	2.756 (4)	2.371 (14)
O6	2.911 (4)	4.39 (2)
F4	3.041 (4)	3.124 (17)

Note: in the NaI adduct, Na^+^⋯F4 and Na^+^⋯O6 distances cannot be considered as bond lengths, thus they are only reported for the sake of comparison.

**Fig. 1.**
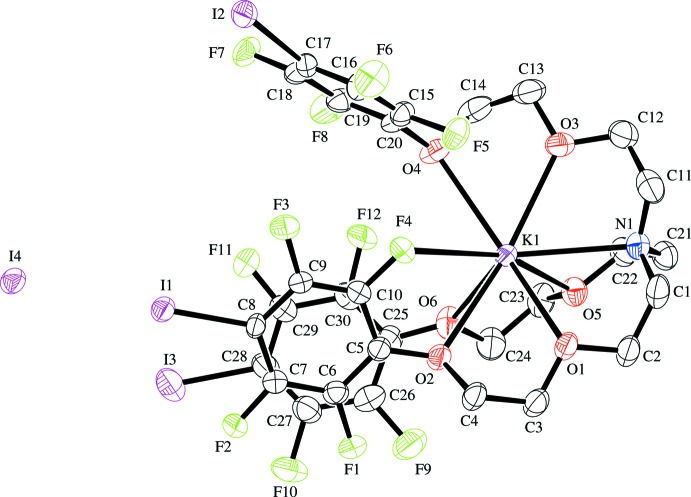
ORTEP-3 view of the salt with numbering scheme. Probability level at 50%.

**Fig. 2.**
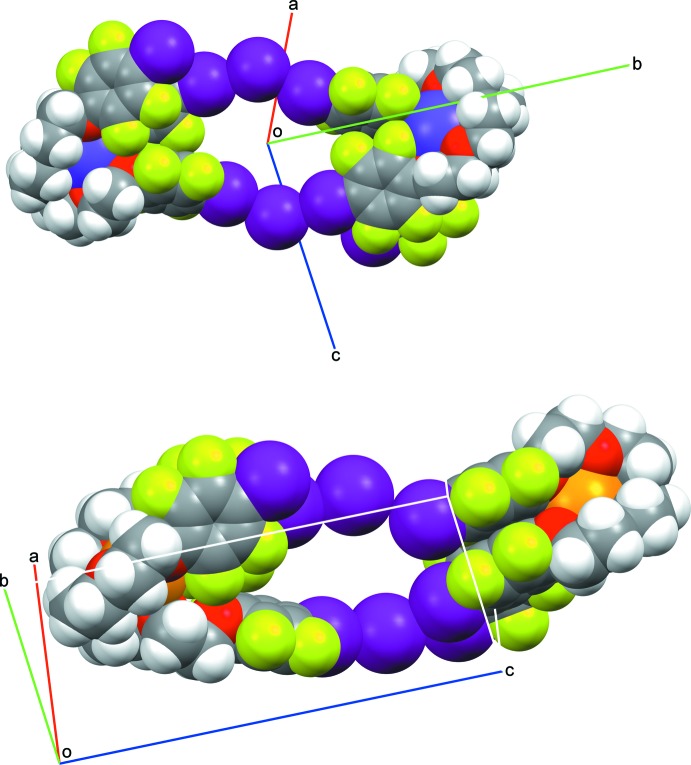
Representation of two receptor/KI and receptor/NaI units (top and bottom, respectively) viewed approximately along the tape direction. The spacefill style evidences how the receptor molecule masks differently the different cations.

**Fig. 3.**
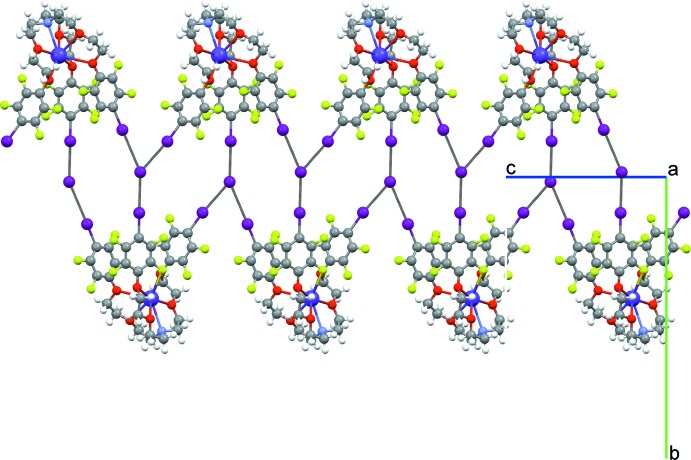
The salt tape viewed along the a-axis (Mercury ball and stick style). Black dashed lines represent the I···I- XBs.

## supplementary crystallographic information

### Comment

Tris(2-(2-(2,3,5,6-tetrafluoro-4-iodophenoxy)ethoxy)ethyl)amine has been systhesized as neutral ditopic receptor for alkali metal halide such as NaI, Mele *et al.*,(2005). It has been also employed as receptor for hydrogen halide systems such as HI, Abate *et al.*,(2009). Here the supramolecular cations (Fig. 1) formed by potassium coordination are linked by two short (namely strong) and one long (namely week) C—I···I^-^ XBs (see Table A) and unlimited tapes are formed. A similar interactions pattern is observed in the isomorphous NaI adduct. In both structures the cation is hosted in a cavity formed by the podand's arms which presents a closed conformation wherein two tetrafluoroiodobenzene rings strictly parallel and the third ring nearly orthogonal. In this system the three C—I bonds are only slightly divergent from each other. The small Na^+^ cation is much more masked than the larger K^+^ one (see Fig. 2). Table B reports some characteristic of the cavity and evidences the differences between the K^+^ and Na^+^ coordination. Figure 3 shows two projections of the salt tapes. The ditopic receptor/HI adduct presents a completely different pattern of interactions. The podand molecules and iodide anions work as bidentate XB donors and acceptors, respectively. The H^+^ is simply bound to the N atom, the supercation adopts conformation different from adopted on K^+^ and Na^+^ coordination as the three tetrafluoroiodobenzene rings are completely divergent.

### Experimental

The chemical synthesis of the neutral triamine compound was carried on following the procedure reported in Mele *et al.*, (2005). Good crystals were obtained from a CHCl_3_ solution of the KI complex after slow solvent diffusion in a box containing vaseline oil.

### Refinement

Hydrogen atoms were constrained with C—H = 0.97 Å and with *U*_iso_(H) = 1.2 times *U*_eq_(C).

### Crystal data

**Table:**

[K(C_30_H_24_F_12_I_3_NO_6_)]I	*F*(000) = 2392
*M**_r_* = 1269.20	*D*_x_ = 2.077 Mg m^−^^3^
Monoclinic, *P*2_1_/*n*	Mo *K*α radiation, λ = 0.71073 Å
*a* = 13.037 (2) Å	Cell parameters from 8602 reflections
*b* = 23.355 (3) Å	θ = 3.0–22.9°
*c* = 13.787 (2) Å	µ = 3.27 mm^−^^1^
β = 104.83 (3)°	*T* = 295 K
*V* = 4058.0 (11) Å^3^	Prism, colourless
*Z* = 4	0.34 × 0.22 × 0.10 mm

### Data collection

**Table:**

Bruker SMART APEX diffractometer	7254 independent reflections
Radiation source: fine-focus sealed tube	5627 reflections with *I* > 2σ(*I*)
Graphite monochromator	*R*_int_ = 0.024
ω and φ scans	θ_max_ = 25.1°, θ_min_ = 1.8°
Absorption correction: multi-scan (*SADABS*; Bruker, 1998)	*h* = −15→15
*T*_min_ = 0.759, *T*_max_ = 1.000	*k* = −27→27
24956 measured reflections	*l* = −16→16

### Refinement

**Table:**

Refinement on *F*^2^	Primary atom site location: structure-invariant direct methods
Least-squares matrix: full	Secondary atom site location: difference Fourier map
*R*[*F*^2^ > 2σ(*F*^2^)] = 0.041	Hydrogen site location: inferred from neighbouring sites
*wR*(*F*^2^) = 0.119	H-atom parameters constrained
*S* = 1.06	*w* = 1/[σ^2^(*F*_o_^2^) + (0.0729*P*)^2^ + 0.2017*P*], where *P* = (*F*_o_^2^ + 2*F*_c_^2^)/3
7254 reflections	(Δ/σ)_max_ < 0.001
487 parameters	Δρ_max_ = 2.37 e Å^−^^3^
0 restraints	Δρ_min_ = −1.25 e Å^−^^3^

### Special details

**Table:**

Experimental. The sample gave two kinds of crystals: rhombic tablets were suitable for data collection, elongated prisms were always twinned of the first. An attempt to collect data at lower temperature failed, probably due to a phase transition or the crystal cracking.
Geometry. All e.s.d.'s (except the e.s.d. in the dihedral angle between two l.s. planes) are estimated using the full covariance matrix. The cell e.s.d.'s are taken into account individually in the estimation of e.s.d.'s in distances, angles and torsion angles; correlations between e.s.d.'s in cell parameters are only used when they are defined by crystal symmetry. An approximate (isotropic) treatment of cell e.s.d.'s is used for estimating e.s.d.'s involving l.s. planes.
Refinement. Refinement of *F*^2^ against ALL reflections. The weighted *R*-factor *wR* and goodness of fit *S* are based on *F*^2^, conventional *R*-factors *R* are based on *F*, with *F* set to zero for negative *F*^2^. The threshold expression of *F*^2^ > σ(*F*^2^) is used only for calculating *R*-factors(gt) *etc*. and is not relevant to the choice of reflections for refinement. *R*-factors based on *F*^2^ are statistically about twice as large as those based on *F*, and *R*- factors based on ALL data will be even larger.

### Fractional atomic coordinates and isotropic or equivalent isotropic displacement parameters (Å^2^)

**Table:**

	*x*	*y*	*z*	*U*_iso_*/*U*_eq_	
I1	−0.08073 (3)	0.12743 (2)	0.28500 (3)	0.07572 (13)	
I2	0.13900 (4)	0.12647 (2)	−0.10931 (3)	0.09463 (16)	
I3	0.16503 (3)	0.16739 (2)	0.61000 (4)	0.1204 (2)	
I4	−0.11661 (3)	−0.01730 (2)	0.27833 (3)	0.08495 (15)	
K1	0.16101 (8)	0.43196 (4)	0.21349 (8)	0.0665 (3)	
N1	0.1758 (4)	0.54837 (19)	0.1446 (4)	0.0859 (12)	
C1	0.0705 (5)	0.5744 (3)	0.1334 (6)	0.107 (2)	
H1A	0.0767	0.6155	0.1269	0.128*	
H1B	0.0230	0.5603	0.0719	0.128*	
C2	0.0235 (5)	0.5624 (2)	0.2174 (6)	0.102 (2)	
H2A	0.0740	0.5717	0.2803	0.122*	
H2B	−0.0391	0.5859	0.2114	0.122*	
O1	−0.0043 (3)	0.50404 (14)	0.2166 (3)	0.0835 (10)	
C3	−0.0554 (5)	0.4908 (2)	0.2922 (5)	0.0985 (19)	
H3A	−0.1138	0.5171	0.2887	0.118*	
H3B	−0.0058	0.4948	0.3576	0.118*	
C4	−0.0956 (4)	0.4315 (2)	0.2787 (5)	0.0919 (18)	
H4A	−0.1385	0.4236	0.3252	0.110*	
H4B	−0.1394	0.4262	0.2110	0.110*	
O2	−0.0044 (3)	0.39276 (15)	0.2977 (3)	0.0834 (10)	
C5	−0.0284 (4)	0.3356 (2)	0.2927 (4)	0.0726 (13)	
C6	−0.0570 (4)	0.3076 (2)	0.3694 (4)	0.0734 (13)	
C7	−0.0718 (4)	0.2493 (2)	0.3675 (4)	0.0749 (13)	
C8	−0.0584 (4)	0.21674 (18)	0.2873 (4)	0.0660 (12)	
C9	−0.0279 (5)	0.2460 (2)	0.2119 (4)	0.0840 (15)	
C10	−0.0125 (5)	0.3019 (2)	0.2141 (4)	0.0846 (15)	
F1	−0.0699 (3)	0.33766 (13)	0.4484 (2)	0.0941 (9)	
F2	−0.1006 (3)	0.22460 (13)	0.4426 (2)	0.0960 (10)	
F3	−0.0133 (4)	0.21664 (15)	0.1329 (2)	0.1187 (13)	
F4	0.0159 (3)	0.33044 (14)	0.1398 (3)	0.1113 (11)	
C11	0.1926 (7)	0.5433 (3)	0.0455 (5)	0.119 (2)	
H11A	0.2008	0.5815	0.0207	0.143*	
H11B	0.1296	0.5266	0.0014	0.143*	
C12	0.2856 (7)	0.5085 (3)	0.0388 (6)	0.117 (2)	
H12A	0.2861	0.5036	−0.0309	0.140*	
H12B	0.3506	0.5279	0.0733	0.140*	
O3	0.2800 (4)	0.45438 (19)	0.0833 (3)	0.1095 (14)	
C13	0.3483 (6)	0.4146 (3)	0.0573 (6)	0.118 (2)	
H13A	0.4118	0.4336	0.0492	0.142*	
H13B	0.3137	0.3961	−0.0055	0.142*	
C14	0.3756 (5)	0.3728 (3)	0.1370 (6)	0.113 (2)	
H14A	0.4136	0.3915	0.1985	0.136*	
H14B	0.4222	0.3442	0.1201	0.136*	
O4	0.2781 (3)	0.34382 (17)	0.1536 (3)	0.0963 (12)	
C15	0.2464 (4)	0.2974 (2)	0.0956 (4)	0.0717 (13)	
C16	0.1645 (4)	0.2984 (2)	0.0133 (4)	0.0721 (12)	
C17	0.1302 (4)	0.2500 (2)	−0.0425 (4)	0.0773 (13)	
C18	0.1782 (4)	0.1992 (2)	−0.0169 (3)	0.0714 (13)	
C19	0.2617 (5)	0.1968 (2)	0.0674 (3)	0.0774 (14)	
C20	0.2953 (5)	0.2455 (3)	0.1221 (4)	0.0832 (15)	
F5	0.1177 (3)	0.34999 (14)	−0.0142 (3)	0.1009 (10)	
F6	0.0509 (3)	0.25554 (18)	−0.1245 (3)	0.1218 (13)	
F7	0.3134 (3)	0.14754 (15)	0.0946 (2)	0.1095 (12)	
F8	0.3768 (3)	0.24039 (18)	0.2043 (2)	0.1218 (14)	
C21	0.2582 (5)	0.5793 (3)	0.2140 (5)	0.0916 (16)	
H21A	0.2250	0.6062	0.2502	0.110*	
H21B	0.2970	0.6015	0.1757	0.110*	
C22	0.3356 (5)	0.5447 (3)	0.2884 (5)	0.0921 (16)	
H22A	0.3766	0.5213	0.2539	0.111*	
H22B	0.3842	0.5700	0.3339	0.111*	
O5	0.2846 (3)	0.50944 (16)	0.3432 (3)	0.0859 (10)	
C23	0.3522 (5)	0.4853 (3)	0.4272 (5)	0.1031 (19)	
H23A	0.3900	0.5157	0.4698	0.124*	
H23B	0.4041	0.4619	0.4065	0.124*	
C24	0.2962 (6)	0.4501 (3)	0.4854 (5)	0.110 (2)	
H24A	0.3467	0.4347	0.5439	0.132*	
H24B	0.2456	0.4735	0.5083	0.132*	
O6	0.2412 (4)	0.40369 (18)	0.4250 (3)	0.0985 (12)	
C25	0.2263 (5)	0.3547 (2)	0.4710 (4)	0.0802 (14)	
C26	0.1778 (5)	0.3506 (3)	0.5479 (5)	0.1009 (19)	
C27	0.1658 (5)	0.2973 (3)	0.5890 (5)	0.0983 (18)	
C28	0.1908 (5)	0.2488 (3)	0.5517 (4)	0.0932 (17)	
C29	0.2358 (8)	0.2504 (3)	0.4750 (6)	0.148 (3)	
C30	0.2551 (8)	0.3058 (3)	0.4368 (6)	0.138 (3)	
F9	0.1451 (4)	0.3983 (2)	0.5853 (4)	0.1440 (16)	
F10	0.1180 (4)	0.2962 (2)	0.6674 (3)	0.1458 (16)	
F11	0.2663 (6)	0.20565 (18)	0.4352 (5)	0.199 (3)	
F12	0.3054 (6)	0.3040 (2)	0.3641 (4)	0.203 (3)	

### Atomic displacement parameters (Å^2^)

**Table:**

	*U*^11^	*U*^22^	*U*^33^	*U*^12^	*U*^13^	*U*^23^
I1	0.0783 (2)	0.0641 (2)	0.0865 (2)	−0.00030 (15)	0.02418 (18)	−0.00568 (15)
I2	0.1145 (3)	0.0786 (3)	0.0969 (3)	−0.0213 (2)	0.0383 (2)	−0.01458 (19)
I3	0.0933 (3)	0.1262 (4)	0.1488 (4)	0.0136 (3)	0.0440 (3)	0.0550 (3)
I4	0.0860 (3)	0.0669 (2)	0.1078 (3)	−0.01087 (17)	0.0354 (2)	−0.01963 (18)
K1	0.0724 (6)	0.0548 (6)	0.0779 (6)	−0.0008 (5)	0.0294 (5)	−0.0074 (5)
N1	0.094 (3)	0.068 (3)	0.099 (3)	−0.007 (2)	0.031 (3)	0.001 (2)
C1	0.096 (4)	0.067 (4)	0.152 (6)	0.007 (3)	0.023 (4)	0.020 (4)
C2	0.082 (4)	0.060 (3)	0.170 (6)	0.002 (3)	0.044 (4)	0.002 (4)
O1	0.072 (2)	0.0564 (19)	0.124 (3)	0.0006 (17)	0.028 (2)	−0.0066 (19)
C3	0.087 (4)	0.068 (3)	0.149 (6)	0.005 (3)	0.047 (4)	−0.011 (3)
C4	0.077 (3)	0.060 (3)	0.146 (5)	−0.006 (3)	0.042 (4)	−0.014 (3)
O2	0.067 (2)	0.065 (2)	0.128 (3)	−0.0003 (17)	0.042 (2)	−0.006 (2)
C5	0.065 (3)	0.063 (3)	0.097 (4)	−0.002 (2)	0.032 (3)	−0.003 (3)
C6	0.067 (3)	0.077 (3)	0.080 (3)	0.000 (3)	0.025 (2)	−0.007 (3)
C7	0.078 (3)	0.072 (3)	0.080 (3)	0.003 (3)	0.030 (3)	0.000 (3)
C8	0.072 (3)	0.044 (2)	0.083 (3)	0.006 (2)	0.023 (2)	0.005 (2)
C9	0.106 (4)	0.067 (3)	0.097 (4)	0.000 (3)	0.058 (3)	−0.008 (3)
C10	0.108 (4)	0.073 (3)	0.087 (3)	−0.002 (3)	0.052 (3)	0.003 (3)
F1	0.117 (2)	0.083 (2)	0.095 (2)	0.0062 (18)	0.0505 (19)	−0.0158 (16)
F2	0.140 (3)	0.077 (2)	0.0896 (19)	−0.0017 (18)	0.063 (2)	0.0035 (15)
F3	0.186 (4)	0.101 (3)	0.089 (2)	−0.008 (2)	0.071 (2)	−0.0216 (18)
F4	0.159 (3)	0.083 (2)	0.117 (2)	−0.014 (2)	0.082 (2)	0.0027 (18)
C11	0.141 (6)	0.110 (5)	0.108 (5)	−0.026 (5)	0.032 (5)	0.017 (4)
C12	0.148 (6)	0.108 (5)	0.110 (5)	−0.025 (5)	0.060 (5)	−0.015 (4)
O3	0.130 (4)	0.097 (3)	0.125 (3)	−0.013 (3)	0.074 (3)	−0.024 (3)
C13	0.119 (5)	0.115 (6)	0.140 (6)	−0.013 (5)	0.069 (5)	−0.044 (5)
C14	0.082 (4)	0.116 (6)	0.143 (6)	−0.004 (4)	0.033 (4)	−0.063 (5)
O4	0.088 (2)	0.092 (3)	0.114 (3)	0.005 (2)	0.036 (2)	−0.037 (2)
C15	0.072 (3)	0.077 (3)	0.069 (3)	0.014 (3)	0.023 (3)	−0.014 (3)
C16	0.075 (3)	0.067 (3)	0.073 (3)	0.016 (3)	0.016 (3)	0.003 (2)
C17	0.073 (3)	0.074 (3)	0.074 (3)	0.007 (3)	−0.001 (3)	0.002 (3)
C18	0.096 (4)	0.055 (3)	0.069 (3)	−0.002 (3)	0.032 (3)	0.003 (2)
C19	0.104 (4)	0.081 (4)	0.050 (3)	0.032 (3)	0.024 (3)	0.010 (2)
C20	0.093 (4)	0.102 (4)	0.052 (3)	0.023 (3)	0.015 (3)	0.001 (3)
F5	0.094 (2)	0.083 (2)	0.117 (2)	0.0325 (18)	0.0105 (19)	0.0145 (18)
F6	0.104 (3)	0.132 (3)	0.101 (2)	0.015 (2)	−0.025 (2)	−0.005 (2)
F7	0.160 (3)	0.084 (2)	0.083 (2)	0.053 (2)	0.029 (2)	0.0157 (17)
F8	0.121 (3)	0.149 (3)	0.0694 (19)	0.049 (3)	−0.0226 (19)	−0.019 (2)
C21	0.090 (4)	0.081 (4)	0.105 (4)	−0.014 (3)	0.028 (3)	0.000 (3)
C22	0.084 (4)	0.087 (4)	0.105 (4)	−0.017 (3)	0.023 (3)	−0.004 (3)
O5	0.085 (2)	0.081 (2)	0.086 (2)	−0.011 (2)	0.012 (2)	−0.003 (2)
C23	0.099 (4)	0.085 (4)	0.109 (5)	−0.004 (4)	−0.003 (4)	−0.004 (4)
C24	0.141 (6)	0.099 (5)	0.081 (4)	−0.006 (4)	0.012 (4)	−0.002 (3)
O6	0.128 (3)	0.081 (3)	0.071 (2)	−0.012 (2)	−0.003 (2)	−0.004 (2)
C25	0.098 (4)	0.075 (3)	0.063 (3)	0.011 (3)	0.011 (3)	0.012 (3)
C26	0.097 (4)	0.105 (5)	0.101 (4)	0.008 (4)	0.024 (4)	−0.026 (4)
C27	0.092 (4)	0.108 (5)	0.103 (4)	−0.002 (4)	0.039 (4)	0.010 (4)
C28	0.086 (4)	0.104 (5)	0.097 (4)	0.017 (3)	0.038 (3)	0.032 (4)
C29	0.239 (10)	0.095 (5)	0.156 (7)	−0.008 (6)	0.134 (7)	0.005 (5)
C30	0.227 (9)	0.092 (5)	0.130 (6)	−0.015 (5)	0.111 (6)	0.000 (4)
F9	0.154 (4)	0.111 (3)	0.182 (4)	0.007 (3)	0.071 (3)	−0.038 (3)
F10	0.168 (4)	0.166 (4)	0.137 (3)	−0.011 (3)	0.101 (3)	−0.012 (3)
F11	0.340 (8)	0.074 (3)	0.262 (6)	0.025 (4)	0.219 (6)	0.008 (3)
F12	0.381 (8)	0.111 (3)	0.203 (4)	−0.021 (4)	0.230 (6)	−0.012 (3)

### Geometric parameters (Å, º)

**Table:**

I1—C8	2.105 (4)	C12—H12B	0.9700
I2—C18	2.106 (5)	O3—C13	1.395 (8)
I3—C28	2.124 (6)	C13—C14	1.445 (10)
K1—O3	2.707 (4)	C13—H13A	0.9700
K1—O1	2.744 (3)	C13—H13B	0.9700
K1—O5	2.756 (4)	C14—O4	1.509 (8)
K1—O4	2.809 (4)	C14—H14A	0.9700
K1—O2	2.849 (3)	C14—H14B	0.9700
K1—N1	2.903 (4)	O4—C15	1.349 (6)
K1—O6	2.911 (4)	C15—C16	1.344 (7)
K1—F4	3.041 (4)	C15—C20	1.375 (7)
N1—C21	1.438 (7)	C16—F5	1.360 (6)
N1—C11	1.443 (8)	C16—C17	1.375 (7)
N1—C1	1.472 (8)	C17—F6	1.328 (6)
C1—C2	1.470 (9)	C17—C18	1.347 (7)
C1—H1A	0.9700	C18—C19	1.374 (7)
C1—H1B	0.9700	C19—F7	1.337 (6)
C2—O1	1.409 (6)	C19—C20	1.373 (8)
C2—H2A	0.9700	C20—F8	1.346 (6)
C2—H2B	0.9700	C21—C22	1.481 (8)
O1—C3	1.408 (7)	C21—H21A	0.9700
C3—C4	1.474 (7)	C21—H21B	0.9700
C3—H3A	0.9700	C22—O5	1.396 (7)
C3—H3B	0.9700	C22—H22A	0.9700
C4—O2	1.464 (6)	C22—H22B	0.9700
C4—H4A	0.9700	O5—C23	1.383 (7)
C4—H4B	0.9700	C23—C24	1.468 (9)
O2—C5	1.368 (6)	C23—H23A	0.9700
C5—C6	1.374 (7)	C23—H23B	0.9700
C5—C10	1.398 (7)	C24—O6	1.440 (7)
C6—F1	1.341 (5)	C24—H24A	0.9700
C6—C7	1.375 (7)	C24—H24B	0.9700
C7—F2	1.320 (6)	O6—C25	1.346 (6)
C7—C8	1.390 (7)	C25—C30	1.326 (9)
C8—C9	1.385 (7)	C25—C26	1.371 (8)
C9—C10	1.319 (7)	C26—F9	1.342 (7)
C9—F3	1.342 (6)	C26—C27	1.394 (9)
C10—F4	1.352 (6)	C27—C28	1.318 (9)
C11—C12	1.482 (10)	C27—F10	1.379 (7)
C11—H11A	0.9700	C28—C29	1.334 (8)
C11—H11B	0.9700	C29—F11	1.289 (8)
C12—O3	1.415 (8)	C29—C30	1.443 (10)
C12—H12A	0.9700	C30—F12	1.331 (7)
			
C21—N1—C11	113.7 (5)	O4—C14—H14A	109.3
C21—N1—C1	112.3 (5)	C13—C14—H14B	109.3
C11—N1—C1	107.4 (6)	O4—C14—H14B	109.3
C2—C1—N1	113.9 (5)	H14A—C14—H14B	108.0
C2—C1—H1A	108.8	C15—O4—C14	114.3 (4)
N1—C1—H1A	108.8	C16—C15—O4	122.9 (5)
C2—C1—H1B	108.8	C16—C15—C20	117.0 (5)
N1—C1—H1B	108.8	O4—C15—C20	120.0 (5)
H1A—C1—H1B	107.7	C15—C16—F5	117.0 (5)
O1—C2—C1	109.7 (5)	C15—C16—C17	122.1 (5)
O1—C2—H2A	109.7	F5—C16—C17	120.9 (5)
C1—C2—H2A	109.7	F6—C17—C18	121.1 (5)
O1—C2—H2B	109.7	F6—C17—C16	117.9 (5)
C1—C2—H2B	109.7	C18—C17—C16	121.0 (5)
H2A—C2—H2B	108.2	C17—C18—C19	118.2 (5)
C3—O1—C2	112.1 (4)	C17—C18—I2	121.8 (4)
O1—C3—C4	109.5 (5)	C19—C18—I2	119.7 (4)
O1—C3—H3A	109.8	F7—C19—C20	119.4 (5)
C4—C3—H3A	109.8	F7—C19—C18	120.4 (5)
O1—C3—H3B	109.8	C20—C19—C18	120.2 (5)
C4—C3—H3B	109.8	F8—C20—C19	117.5 (5)
H3A—C3—H3B	108.2	F8—C20—C15	121.0 (5)
O2—C4—C3	108.1 (4)	C19—C20—C15	121.5 (5)
O2—C4—H4A	110.1	N1—C21—C22	116.6 (5)
C3—C4—H4A	110.1	N1—C21—H21A	108.2
O2—C4—H4B	110.1	C22—C21—H21A	108.2
C3—C4—H4B	110.1	N1—C21—H21B	108.2
H4A—C4—H4B	108.4	C22—C21—H21B	108.2
C5—O2—C4	115.4 (4)	H21A—C21—H21B	107.3
O2—C5—C6	122.0 (4)	O5—C22—C21	111.2 (5)
O2—C5—C10	120.8 (4)	O5—C22—H22A	109.4
C6—C5—C10	116.8 (5)	C21—C22—H22A	109.4
F1—C6—C5	119.4 (5)	O5—C22—H22B	109.4
F1—C6—C7	119.2 (4)	C21—C22—H22B	109.4
C5—C6—C7	121.4 (5)	H22A—C22—H22B	108.0
F2—C7—C6	119.0 (4)	C23—O5—C22	113.8 (5)
F2—C7—C8	120.2 (5)	O5—C23—C24	112.7 (6)
C6—C7—C8	120.8 (5)	O5—C23—H23A	109.0
C9—C8—C7	116.5 (4)	C24—C23—H23A	109.0
C9—C8—I1	122.9 (4)	O5—C23—H23B	109.0
C7—C8—I1	120.6 (3)	C24—C23—H23B	109.0
C10—C9—F3	118.2 (5)	H23A—C23—H23B	107.8
C10—C9—C8	122.8 (5)	O6—C24—C23	110.5 (5)
F3—C9—C8	119.0 (5)	O6—C24—H24A	109.6
C9—C10—F4	122.7 (5)	C23—C24—H24A	109.6
C9—C10—C5	121.6 (5)	O6—C24—H24B	109.6
F4—C10—C5	115.7 (5)	C23—C24—H24B	109.6
N1—C11—C12	115.4 (6)	H24A—C24—H24B	108.1
N1—C11—H11A	108.4	C25—O6—C24	118.5 (4)
C12—C11—H11A	108.4	C30—C25—O6	118.5 (5)
N1—C11—H11B	108.4	C30—C25—C26	116.4 (6)
C12—C11—H11B	108.4	O6—C25—C26	125.0 (6)
H11A—C11—H11B	107.5	F9—C26—C25	119.7 (7)
O3—C12—C11	109.2 (6)	F9—C26—C27	120.4 (6)
O3—C12—H12A	109.8	C25—C26—C27	119.9 (6)
C11—C12—H12A	109.8	C28—C27—F10	119.8 (6)
O3—C12—H12B	109.8	C28—C27—C26	123.0 (6)
C11—C12—H12B	109.8	F10—C27—C26	117.0 (6)
H12A—C12—H12B	108.3	C27—C28—C29	119.2 (7)
C13—O3—C12	112.0 (5)	C27—C28—I3	122.8 (4)
O3—C13—C14	107.6 (6)	C29—C28—I3	118.0 (6)
O3—C13—H13A	110.2	F11—C29—C28	124.2 (7)
C14—C13—H13A	110.2	F11—C29—C30	117.9 (6)
O3—C13—H13B	110.2	C28—C29—C30	117.9 (7)
C14—C13—H13B	110.2	C25—C30—F12	122.2 (6)
H13A—C13—H13B	108.5	C25—C30—C29	123.4 (6)
C13—C14—O4	111.5 (6)	F12—C30—C29	114.4 (7)
C13—C14—H14A	109.3		
			
C21—N1—C1—C2	−76.9 (6)	F6—C17—C18—C19	178.3 (5)
C11—N1—C1—C2	157.3 (6)	C16—C17—C18—C19	0.7 (8)
N1—C1—C2—O1	−69.4 (7)	F6—C17—C18—I2	4.5 (7)
C1—C2—O1—C3	−176.6 (5)	C16—C17—C18—I2	−173.1 (4)
C2—O1—C3—C4	172.1 (5)	C17—C18—C19—F7	−178.5 (5)
O1—C3—C4—O2	66.8 (7)	I2—C18—C19—F7	−4.5 (6)
C3—C4—O2—C5	177.1 (5)	C17—C18—C19—C20	−1.1 (7)
C4—O2—C5—C6	−77.6 (6)	I2—C18—C19—C20	172.9 (4)
C4—O2—C5—C10	110.0 (6)	F7—C19—C20—F8	−3.1 (7)
O2—C5—C6—F1	5.2 (7)	C18—C19—C20—F8	179.5 (4)
C10—C5—C6—F1	177.8 (5)	F7—C19—C20—C15	178.4 (5)
O2—C5—C6—C7	−174.2 (5)	C18—C19—C20—C15	1.0 (8)
C10—C5—C6—C7	−1.5 (8)	C16—C15—C20—F8	−178.9 (5)
F1—C6—C7—F2	1.5 (8)	O4—C15—C20—F8	−1.7 (8)
C5—C6—C7—F2	−179.2 (5)	C16—C15—C20—C19	−0.4 (8)
F1—C6—C7—C8	−179.5 (4)	O4—C15—C20—C19	176.8 (5)
C5—C6—C7—C8	−0.2 (8)	C11—N1—C21—C22	−103.3 (6)
F2—C7—C8—C9	−179.9 (5)	C1—N1—C21—C22	134.5 (6)
C6—C7—C8—C9	1.2 (8)	N1—C21—C22—O5	−55.2 (7)
F2—C7—C8—I1	−1.2 (7)	C21—C22—O5—C23	−167.0 (5)
C6—C7—C8—I1	179.8 (4)	C22—O5—C23—C24	178.1 (5)
C7—C8—C9—C10	−0.4 (9)	O5—C23—C24—O6	60.2 (8)
I1—C8—C9—C10	−179.0 (5)	C23—C24—O6—C25	150.9 (6)
C7—C8—C9—F3	179.6 (5)	C24—O6—C25—C30	−128.5 (8)
I1—C8—C9—F3	1.0 (8)	C24—O6—C25—C26	55.2 (9)
F3—C9—C10—F4	0.8 (9)	C30—C25—C26—F9	−179.0 (7)
C8—C9—C10—F4	−179.2 (6)	O6—C25—C26—F9	−2.7 (10)
F3—C9—C10—C5	178.6 (6)	C30—C25—C26—C27	3.0 (10)
C8—C9—C10—C5	−1.4 (10)	O6—C25—C26—C27	179.3 (6)
O2—C5—C10—C9	175.0 (5)	F9—C26—C27—C28	175.9 (6)
C6—C5—C10—C9	2.3 (9)	C25—C26—C27—C28	−6.1 (11)
O2—C5—C10—F4	−7.0 (8)	F9—C26—C27—F10	0.9 (10)
C6—C5—C10—F4	−179.8 (5)	C25—C26—C27—F10	178.9 (6)
C21—N1—C11—C12	65.8 (7)	F10—C27—C28—C29	179.2 (7)
C1—N1—C11—C12	−169.3 (6)	C26—C27—C28—C29	4.3 (12)
N1—C11—C12—O3	52.8 (8)	F10—C27—C28—I3	−1.9 (9)
C11—C12—O3—C13	164.5 (6)	C26—C27—C28—I3	−176.8 (5)
C12—O3—C13—C14	153.4 (6)	C27—C28—C29—F11	177.7 (9)
O3—C13—C14—O4	58.0 (7)	I3—C28—C29—F11	−1.2 (14)
C13—C14—O4—C15	87.5 (6)	C27—C28—C29—C30	0.1 (13)
C14—O4—C15—C16	−101.9 (6)	I3—C28—C29—C30	−178.8 (7)
C14—O4—C15—C20	81.1 (7)	O6—C25—C30—F12	5.3 (13)
O4—C15—C16—F5	4.4 (7)	C26—C25—C30—F12	−178.2 (8)
C20—C15—C16—F5	−178.5 (4)	O6—C25—C30—C29	−175.2 (8)
O4—C15—C16—C17	−177.0 (5)	C26—C25—C30—C29	1.4 (13)
C20—C15—C16—C17	0.1 (8)	F11—C29—C30—C25	179.2 (9)
C15—C16—C17—F6	−177.8 (5)	C28—C29—C30—C25	−3.1 (15)
F5—C16—C17—F6	0.6 (7)	F11—C29—C30—F12	−1.2 (14)
C15—C16—C17—C18	−0.2 (8)	C28—C29—C30—F12	176.5 (9)
F5—C16—C17—C18	178.2 (5)		

### Table A. I···I^-^ XBs and short F···F contacts (Å and °). For the sake of comparison, a Cambridge Structural Database (Allen, 2002) search for C—I···I^-^ contacts gives 109 hits with a mean I···I^-^ distance of 3.53 (3) Å, the lower and higher quartiles being 3.43 and 3.63 Å, respectively.

**Table:**

C—X···Y	X···Y	C-X···Y
C8—I1···I4	3.4106 (6)	179.30 (13)
C8—I2···I4^i^	3.4157 (6)	168.45 (13)
C8—I3···I4^ii^	3.9437 (7)	179.21 (18)

Symmetry codes: (i) -x, -y, -z; (ii) -x, -y, -z+1.

### Table B. Short contacts in receptor–metal cation system for KI and NaI adducts.

**Table:**

	K^+^	Na^+^
N1	2.903 (4)	2.515 (15)
O1	2.744 (3)	2.388 (12)
O2	2.849 (3)	2.716 (12)
O3	2.707 (4)	2.463 (13)
O4	2.809 (4)	2.424 (12)
O5	2.756 (4)	2.371 (14)
O6	2.911 (4)	4.39 (2)
F4	3.041 (4)	3.124 (17)

In the NaI adduct, Na^+^···F4 and Na^+^···O6 distances cannot be considered as bond lengths, thus they are only reported for sake of comparison.

## References

[bb1] Abate, A., Biella, S., Cavallo, G., Meyer, F., Neukirch, H., Metrangolo, P., Pilati, T., Resnati, G. & Terraneo, G. (2009). *J. Fluorine Chem.* **130**, 1171–1177.

[bb2] Allen, F. H. (2002). *Acta Cryst.* B**58**, 380–388. 10.1107/s0108768102003890 12037359

[bb3] Bruker (1998). *APEX2*, *SAINT* and *SADABS* Bruker AXS Inc., Madison, Wisconsin, USA.

[bb4] Burla, M. C., Camalli, M., Carrozzini, B., Cascarano, G. L., Giacovazzo, C., Polidori, G. & Spagna, R. (2003). *J. Appl. Cryst.* **36**, 1103.

[bb5] Farrugia, L. J. (2012). *J. Appl. Cryst.* **45**, 849–854.

[bb6] Macrae, C. F., Edgington, P. R., McCabe, P., Pidcock, E., Shields, G. P., Taylor, R., Towler, M. & van de Streek, J. (2006). *J. Appl. Cryst.* **39**, 453–457.

[bb7] Mele, A., Metrangolo, P., Neukirch, H., Pilati, T. & Resnati, G. (2005). *J. Am. Chem. Soc.* **127**, 14972–14973. 10.1021/ja054862h 16248605

[bb8] Sheldrick, G. M. (2012). *SHELXL2012* University of Göttingen, Germany.

